# Monitored Tomographic Reconstruction—An Advanced Tool to Study the 3D Morphology of Nanomaterials

**DOI:** 10.3390/nano11102524

**Published:** 2021-09-27

**Authors:** Konstantin Bulatov, Marina Chukalina, Kristina Kutukova, Vlad Kohan, Anastasia Ingacheva, Alexey Buzmakov, Vladimir V. Arlazarov, Ehrenfried Zschech

**Affiliations:** 1 Federal Research Center “Computer Science and Control” of Russian Academy of Sciences, 117312 Moscow, Russia; k.bulatov@smartengines.com (K.B.); vva@smartengines.com (V.V.A.); 2 Smart Engines Service LLC, 117312 Moscow, Russia; v.kokhan@smartengines.com (V.K.); a.ingacheva@smartengines.com (A.I.); a.buzmakov@smartengines.com (A.B.); 3 Federal Scientific Research Center “Crystallography and Photonics” of Russian Academy of Sciences, 119333 Moscow, Russia; 4 Fraunhofer Institute for Ceramic Technologies and Systems, 01109 Dresden, Germany; 5 Institute for Information Transmission Problems of Russian Academy of Sciences (Kharkevich Institute), 127051 Moscow, Russia; 6 DeepXscan GmbH, 01109 Dresden, Germany; ehrenfried.zschech@deepxscan.com

**Keywords:** nano X-ray computed tomography, hierarchical structures, 3D morphology, monitored reconstruction, stopping rule, time reducing, quality metrics

## Abstract

Detailed and accurate three-dimensional (3D) information about the morphology of hierarchically structured materials is derived from multi-scale X-ray computed tomography (XCT) and subsequent 3D data reconstruction. High-resolution X-ray microscopy and nano-XCT are suitable techniques to nondestructively study nanomaterials, including porous or skeleton materials. However, laboratory nano-XCT studies are very time-consuming. To reduce the time-to-data by more than an order of magnitude, we propose taking advantage of a monitored tomographic reconstruction. The benefit of this new protocol for 3D imaging is that the data acquisition for each projection is interspersed by image reconstruction. We demonstrate this new approach for nano-XCT data of a novel transition-metal-based materials system: MoNi_4_ electrocatalysts anchored on MoO_2_ cuboids aligned on Ni foam (MoNi_4_/MoO_2_@Ni). Quantitative data that describe the 3D morphology of this hierarchically structured system with an advanced electrocatalytically active nanomaterial are needed to tailor performance and durability of the electrocatalyst system. We present the framework for monitored tomographic reconstruction, construct three stopping rules for various reconstruction quality metrics and provide their experimental evaluation.

## 1. Introduction

Multi-scale X-ray computed tomography (XCT) is an efficient approach to study the 3D morphology of natural or engineered hierarchically structured systems and materials [1]. This approach combines 3D–image information at several hierarchical levels, characterized by typical feature sizes of structures, to fully resolve the 3D morphology of complex systems. Experimentally, this approach requires the combination of several XCT techniques with different sample volumes and resolutions, including nano-XCT. Measurement times for complete XCT experiments depend strongly on the brightness of the X-ray source. The brightness of laboratory X-ray sources is typically several orders of magnitude lower than that of synchrotron radiation sources. Since X-ray microscopes with sub-100 nm resolution, as needed for the characterization of nanostructures and nano-scale materials, include X-ray optics with limited efficiency, data acquisition times for full tomographies are long, particularly for laboratory tools [2]. As a consequence, additional approaches are needed, including advanced data analysis and new protocols for image data acquisition (“design of experiment”). One option to reduce time-to-data for nano-XCT studies of nanomaterials is the application of advanced data analysis procedures. Since artificial neural networks are able to train themselves to recognize complex patterns, deep learning algorithms have been applied to reduce image artifacts and to handle missing data in XCT studies. Mapping filtered back projections identically and building up a deep neural network architecture allows for the addition of different compensation layers for several types of artifacts through end-to-end training [3]. As an example, missing data artifacts, e.g., from a limited-angle tomography, were reduced by trained convolutional neural networks. This approach extracts and suppresses implicit features of artifacts from reconstructed images [4]. Neural networks were also successfully used for noise reduction. While the expected internal structure information of an object is retained, shorter image acquisition times are achieved [5,6]. What all these approaches have in common is that the deep neural network is added as a post-correction step subsequently to the reconstruction step.

A tomographic reconstruction methodology with incorporated artificial-intelligence (AI) algorithms was described by Topal et al. [7]. This approach considers unavoidable misalignment and object motion during high-resolution 3D data acquisition, demonstrating that high-quality 3D images could be obtained, and data from incomplete data sets can be recovered. A novel computer vision methodology combined with a deep neural network approach provides estimates for motion by tracking features through adjacent projections. This tomographic–reconstruction algorithm is empowered by sophisticated correction modules that autonomously estimate and effectively suppress artifacts and random errors using gradient descent and deep learning algorithms (examples see [8,9]). Another development to shorten the data acquisition time for XCT was reported by Bulatov et al. [10]. A monitored reconstruction process was proposed that performs the reconstruction procedure not only after the completion of the measurement protocol. That means, this monitored tomographic reconstruction can be considered as an “anytime algorithm”. The acquisition of the individual two-dimensional (2D) projections is interspersed by image reconstruction, with the final stop of the projection data acquisition occurring according to a defined stopping rule.

In this paper, we are applying monitored tomographic reconstruction to the analysis of nano-XCT data of a complex hierarchically structured materials system, demonstrating the potential of this new approach for the generation of detailed and accurate 3D information about the morphology of advanced nanomaterials in a significantly shorter period of time. The chosen example, a high performance and durable electrocatalytic system with a tailored morphology, is a novel transition-metal-based materials system for fast water dissociation: MoNi4 electrocatalysts anchored on MoO_2_ cuboids aligned on Ni foam (MoNi_4_/MoO_2_@Ni) [11]. XCT, both nano-XCT and micro-XCT, are suitable techniques to provide the morphology of the (sub-)structures of such hierarchically structured materials and systems [8]. Since individual projections, i.e., 2D slices of the investigated object, include very different individual features, we used several different 2D slices of one object for evaluation. The monitored tomographic reconstruction and its application to nano-XCT data of nanomaterials are presented in this paper, with stopping rules discussed in detail. Finally, the plausibility of the obtained results is provided.

## 2. Materials and Methods

### 2.1. Synthesis and Characterization of the MoNi_4_/MoO_2_@Ni Material System

Highly performant and durable systems for electrochemical energy storage and conversion require the design and synthesis of materials with optimized hierarchical 3D morphology and surface structures with high activity. In addition to the intrinsic properties of the constituent components that determine chemical activity and conductivity, the material’s 3D morphology is crucial to prevent agglomeration of particles and to promote mass transfer (i.e., reactant diffusion and product release) [8]. The design and synthesis of robust 3D structures, e.g., electrocatalysts, with an optimized hierarchical morphology, are of particular interest for the upscaling of processes and for the development of technologies to fabricate advanced energy conversion devices. They will therefore play an increasing role in the future.

A novel transition-metal-based materials system for fast water dissociation, MoNi_4_ electrocatalysts anchored on MoO_2_ cuboids aligned on Ni foam (MoNi_4_/MoO_2_@Ni), was selected for this study. Mo-Ni-based alloy (Mo_x_Ni_y_) electrocatalysts provide high electrocatalytic efficiency for the hydrogen evolution reaction (HER).

The synthesis of the MoNi_4_ electrocatalyst was performed in a two-step process. Firstly, NiMoO_4_ cuboids were grown on a piece of Ni foam (1 × 3 cm^2^) during a hydrothermal reaction at 150 °C for 6 h in 15 mL deionized water containing Ni(NO_3_)_2_·6H_2_O (0.04 M) and (NH_4_)_6_Mo_7_O_24_·4H_2_O (0.01 M). Subsequently, while the as-synthesized NiMoO_4_ cuboids were heated in a H_2_/Ar (*v*/*v*, 5/95) atmosphere at 500 °C for 2 h, Ni atoms were diffused in a controlled way to the surface of the cuboid, forming numerous MoNi_4_ nanoparticles with a size of 20–100 nm on the surface of the MoO_2_ cuboids. Simultaneously, Ni out-diffusion caused a change in the composition of the cuboids from NiMoO_4_ to MoO_2_. The MoNi_4_ electrocatalyst exhibited a zero onset overpotential, an overpotential of 15 mV at 10 mAcm^−2^ and a low Tafel slope of 30 mV per decade in 1 M KOH electrolyte [11].

Micro- and nano-XCT, scanning electron microscopy (SEM), and transition electron microscopy (TEM) were applied to image the electrocatalytically active nano-sized MoNi_4_ particles anchored on MoO_2_ micro cuboids vertically aligned on the conductive Ni foam [8,11]. The needle-like MoO_2_ micro cuboids, which were vertically arranged on the Ni foam, had rectangular cross-sections of 0.5 × 1 μm^2^ and 10 to 20 μm in length. MoNi_4_ nanoparticles 20 to 100 nm in size were positioned on the surface of the MoO_2_ cuboids [8].

### 2.2. Nano-XCT (Laboratory Tool)

The design of 3D electrocatalysts with high performance and durability requires both an optimized hierarchical morphology and surface structures with high activity. A systematic approach to determine the morphology of hierarchically structured materials with high accuracy is based on multi-scale X-ray tomography since it provides 3D information non-destructively at several length scales, with a reasonable compromise between the considered sample volume and spatial resolution [1]. Here, we report the nano-XCT study of MoNi_4_/MoO_2_@Ni to image the MoNi_4_ nanoparticles located on MoO_2_ micro cuboids, since unavoidable misalignment and thermomechanical effects are more critical for X-ray imaging with high spatial resolution.

Three-dimensional tomographic images of an object are generated by acquiring many nondestructively obtained radiographs (2D projection images) of an object at several projection angles. Subsequently, a reconstruction algorithm is applied to these images for the nano-XCT technique with sub-100 nm resolution. It is necessary but difficult in practice, if not impossible, to align the various system components with sub-micron precision. Therefore, the acquired projections usually do not strictly match the acquisition geometry as defined by the reconstruction algorithm. This mismatch results in artifacts in the reconstructed images that can seriously degrade their quality. Consequently, it is critical to explain the root causes of the artifacts in order to understand what can be done to avoid or mitigate them. Image reconstruction could be a powerful method for addressing the issues of accuracy of the XCT technique.

**Figure 1:**
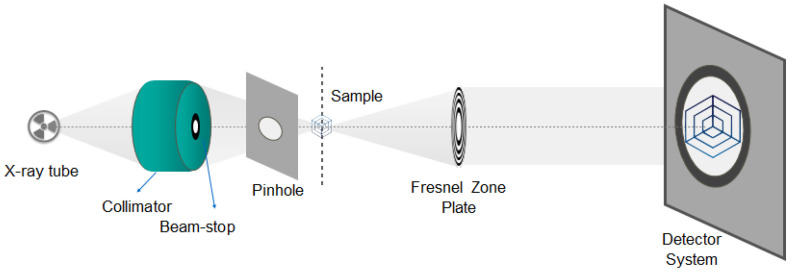
Characteristic early parallel-beam geometry for a nano-XCT setup.

The nano-XCT data acquisition was performed using an Xradia nano-XCT 100 tool (Concord, CA, USA), equipped with a rotating anode X-ray source (Cu-Kα radiation, 8 keV photon energy). Figure 1 shows the scheme of the X-ray microscope used [8]. The X-ray microscopy setup offers (nearly) parallel-beam imaging. Therefore, an angular range of 180° is sufficient for the acquisition of the tomography data. Laboratory X-ray microscopes operating in the multi-keV range use Fresnel zone plates as focusing lenses, resulting in a spatial resolution of the microscope of sub-100 nm. The enlarged image is projected onto a thin scintillator and subsequently recorded by an optical microscope with a CCD camera (Crytur, Turnov, Czech Republic).

A sample with MoO_2_ cuboids, each of them with a size of about 35 × 35 × 35 µm^3^, was picked up from the Ni foam and fixed on the tip of a tungsten wire. The laboratory nano-XCT studies were performed in standard resolution mode (40× FZP magnification × 20× optical magnification = 800× total magnification). The field of view width and height were 65 µm with 1024 pixels for each, resulting in a voxel size of 65 nm. The tilt series for the tomography consisted of 801 images within an angular range of 180°. The exposure time per image was 180 s.

**Figure 2:**
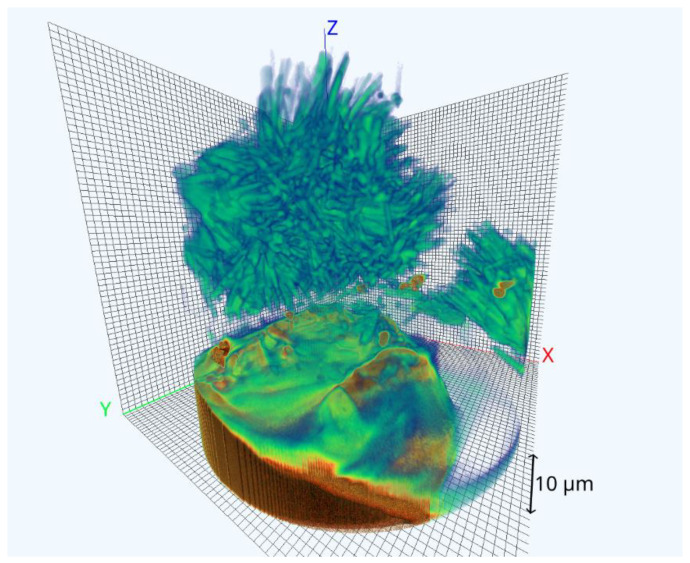
Reconstructed volume from all collected projections.

We used Smart Tomo Engine software [12] to reconstruct the 3D numerical image of the investigated structure. The result of the 3D reconstruction from the fully collected set of projections is presented in Figure 2. A Filtered Back Projection algorithm was used for reconstruction [13].

## 3. Monitored Reconstruction

It is normal practice that tomographic image reconstruction starts after the completion of the scan protocol, after which the recognition process can be started [14]. In this section, we turn to the monitored reconstruction approach introduced in [10] in order to explain how to look at the reconstruction as a process with the monitored result, i.e., we present a framework for monitored reconstruction. The fact that the quality of reconstruction depends not only on the number of projections but also on the sequence of their shooting was already shown in the 1990s [15]. In our modeling experiment, we transferred the projections for reconstruction in some random sequences. Below, we consider different XY slices of the reconstructed object (Figure 2) as independent samples. The object itself is at the top of the image and has a needle-like shape. Below is the sample holder. Several gold particles used for calibration are placed at the bottom of the object (brown color).

### 3.1. Cost of the Collected Projections

We collected a sequence of projections $X=\left(X_{1},X_{2},\ldots \right)$ from the slice θ∈Θ according to a protocol. We can consider $\chi_{i}$ as a random vector that depends on the slice θ and is in charge of the projection angle. $R_{n}\left(x_{1},x_{2},\ldots ,x_{n}\right)$ is the slice reconstructed from *n* collected projections. $\epsilon \left(R_{n}\left(x_{1},\ldots ,x_{n}\right),\theta \right)$ is the reconstruction error for the slice θ. Our task was to obtain a reconstruction result with the lowest value of the error $\epsilon \left(R_{n}\left(x_{1},\ldots ,x_{n}\right),\theta \right)$. We assumed that the value of $\epsilon \left(R_{n}\left(x_{1},\ldots ,x_{n}\right),\theta \right)$ decreases over time.

Projection measurement has a cost in terms of measurement time, ignoring computation time. The best compromise between the current error and cost of the collected projections leads to the problem of optimal stopping. We had to determine the last projection when the collecting of projections should be terminated. In that way, the current result of reconstruction would be final. A comprehensive description of optimal stopping problems formalization can be found in [15,16].

We denote the sequence of cost functions by $c=\left(c_{0},c_{1}\left(x_{1}\right),c_{2}\left(x_{1},x_{2}\right),\ldots \right)$, $c_{n}\left(x_{1},\ldots ,x_{n}\right)<c_{n+1}\left(x_{1},\ldots ,x_{n},x_{n+1}\right)$. The cost function $c_{n}$ has a domain $\chi^{n}$, denoting the total cost of projection collecting $X_{1}=x_{1},\ldots ,X_{n}=x_{n}$. The total loss $L_{n}\left(x_{1},\ldots ,x_{n}\right)$ of collecting n projections and reconstruction $R_{n}\left(x_{1},\ldots ,x_{n}\right)$ is a sum of the reconstruction error and the cost of required projections:

(1) $$\begin{array}{c} L_{n}\left(x_{1},\ldots ,x_{n}\right)=\\ =\epsilon \left(R_{n}\left(x_{1},\ldots ,x_{n}\right),\theta \right)+c_{n}\left(x_{1},\ldots ,x_{n}\right). \end{array}$$

**Figure 3:**
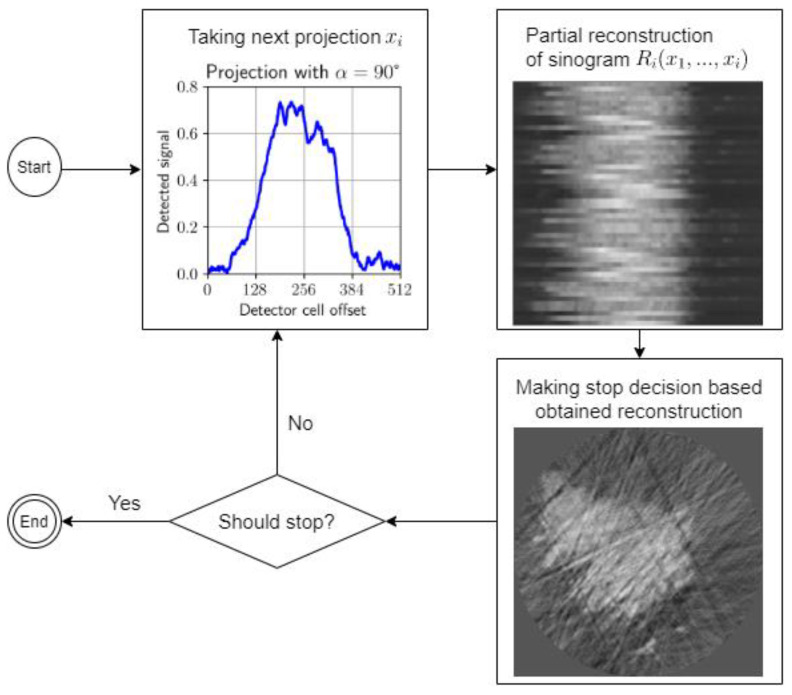
A schematic plot of the monitored reconstruction process.

The problem of stopping data collection and calculations involves choosing a stopping rule, which should optimize the expected loss. Figure 3 presents a schematic plot of the monitored reconstruction process.

### 3.2. Absolute and Relative Estimation Errors

To evaluate the accuracy of the partial reconstruction results, we used the absolute estimation errors (2) in terms of $\ell_{2}$ norm:

(2) $$\mathrm{RSRE}\left(R_{n},\theta \right)=∥R_{n}-R^{*}\left(\theta \right){∥}_{2}$$

and two relative estimation errors in terms of $\ell_{2}$ norm. The first one is normalized to a slice reconstruction from the full set of projections $R^{*}\left(\theta \right):$

(3) $$NRSRE\left(R_{n},\theta \right)=\frac{∥R_{n}-R^{*}\left(\theta \right){∥}_{2}}{∥R^{*}\left(\theta \right)∥}$$

The second relative estimation error is normalized to the sum of pixel values of the slice reconstructed from the complete set of projections $S\left(R^{*}\left(\theta \right)\right):\;$

(4) $$S-\mathrm{RSRE}\left(R_{n},\theta \right)=\frac{∥R_{n}-R^{*}\left(\theta \right){∥}_{2}}{S\left(R^{*}\left(\theta \right)\right)}$$

Here $R_{n}$ is a current partial reconstruction of the slice. The RSRE metric is a traditional way to compare two images, which can be used for matching the reconstructed images. The use of normalizations is due to the appearance of various artifacts on tomographic images. In addition, *S*-RSRE errors are sensitive to the spectral width of the probe.

In general, the tomographic imaging process takes the number of projections for reconstruction that are known in advance. In the case of the monitored reconstruction, we accept that the data acquisition protocol defines a natural stopping point where all required projections are acquired, e.g., at the stage n=T. The final reconstruction result $R_{T}$ corresponsds to all collected projections. To prove the impact of the stopping rules, we calculate the error component associated with the number of collected projections. To conclude, it is sufficient to use the last reconstruction result $R_{T}$ instead of the ground truth value $R^{*}\;$ for the stopping problem. Consequently, the obtained reconstruction result corresponds to that which can be achieved using the measurement protocol for the zero value of the error function.

### 3.3. Stopping Rules

We define a stopping rule ϕ as a sequence of functions in which each function is the conditional probability of stopping data acquisition, given that n projections have been collected and $X_{1}=x_{1},\ldots ,X_{n}=x_{n}$ [17]. With a selected stopping rule ϕ, an arbitrary variable N can be specified, which represents the corresponding stopping time. The stopping time N and stopping rule ϕ are associated in this way:

(5) $$\begin{array}{c} \phi_{n}\left(x_{1},\ldots ,x_{n}\right)=\\ =P\left(N=n\;|\;N\geq n,X_{1}=x_{1},\ldots ,X_{n}=x_{n}\right). \end{array}$$

The stopping problem requires choosing a proper stopping rule ϕ that would minimize the expected loss function V(ϕ), which can be defined as follows

(6) $$V\left(\phi \right)=E\left(\overset{=\infty }{\underset{n=0}{\sum }}(\phi_{n}\left(X_{1},\ldots ,X_{n}\right)\overset{n-1}{\underset{i=0}{\prod }}(1-\phi_{i}\left(X_{1},\ldots ,X_{i}\right)))L_{n}\left(X_{1},\ldots ,X_{n}\right)\right)$$

where the “=∞” assumes that the summation overvalues of n from 0 to ∞, including ∞. In terms of the arbitrary stopping time N, the expected loss can be defined as follows

(7) $$V\left(\phi \right)=E\left(L_{N}\left(X_{1},\ldots ,X_{N}\right)\right).$$

Solving the stopping Equation (6) determines the moment when the quality of the reconstructed image is good enough given the expended cost, i.e., when the process of monitored reconstruction should be stopped. The scheme of the monitored reconstruction model is presented in Figure 3.

If we assume a moment n=T where the process must stop in any case, independent of the reconstruction accuracy, the problem can be defined as a finite-horizon-stopping problem. For the known distributions of $X_{1},X_{2},\ldots$ and total losses $L_{n}$ (1), the general method for finding an optimal value for a stopping rule is backward induction [18,19]. Monotone-stopping problems [18,20] are a particular case. Let $A_{n}$ denote the event $\left\{L_{n}\leq E_{n}\left(L_{n+1}\right)\right\}$. The optimal stopping problem is defined as monotone if $\forall n\geq 0:A_{n}\subset A_{n+1}$. Here, $E_{n}\left(\cdot \right)$ is the conditional expectation of a random variable given that the first n observations are taken. If the stopping problem is monotonic, an optimal stopping rule can be found in form [10]:

(8) $$N=\min\left\{n\geq 0:L_{n}\leq E_{n}\left(L_{n+1}\right)\right\}.$$

With the loss function (1), the rule takes the following form:

(9) $$\begin{array}{c} N=\min\{n\geq 0:\epsilon \left(R_{n},\theta \right)-E_{n}\left(\epsilon \left(R_{n+1},\theta \right)\right)\leq \\ \leq E_{n}\left(c_{n+1}\right)-c_{n}\}. \end{array}$$

In our experiments, we used $\epsilon \left(R_{n},\theta \right)=\rho \left(R_{n},R^{*}\left(\theta \right)\right)$ in terms of (6). Furthermore, the stopping rules can be rewritten:

(10) $$\begin{array}{c} N_{\Delta }^{\mathrm{RSRE}}=\min\{n\geq 0:E_{n}\left(∥R_{n}-R_{n+1}{∥}_{2}\right)\leq \\ \leq E_{n}\left(c_{n+1}\right)-c_{n}\}.\\ N_{\Delta }^{\mathrm{NRSRE}}=\min\{n\geq 0:E_{n}\left(∥R_{n}-R_{n+1}{∥}_{2}\right)\leq \\ \leq ∥R_{T}{∥}_{2}\cdot \left(E_{n}\left(c_{n+1}\right)-c_{n}\right)\},\\ N_{\Delta }^{S-\mathrm{RSRE}}=\min\left\{n\geq 0:E_{n}\left(|R_{n}-R_{n+1}{|}_{2}\right)\leq S\left(R_{T}\right)\cdot \left(E_{n}\left(c_{n+1}\right)-c_{n}\right)\right\}. \end{array}$$

To apply the stopping rules, we used the straightforward-estimation method [10], assuming that the looking distance is close to the distance between the two most recently obtained results:

(11) $$E_{n}\left(∥R_{n}-R_{n+1}{∥}_{2}\right)\approx ∥R_{n-1}-R_{n}{∥}_{2}.$$

Since the measurement time of one projection (exposition time) does not change during the experiment and is long compared to the time spent on reconstruction, the cost function is relative to the number of acquired projections. The looking value of cost function on the next step in the process is larger than the current cost function on the constant value c:

(12) $$E_{n}\left(c_{n+1}\right)-c_{n}=c.$$

To implement the stopping rule NRSRE, the following dependence model between the reconstruction result norm and the stage number n can be written:

(13) $${∥R_{n}∥}_{2}\approx \frac{a_{0}}{a_{1}+n}+a_{2}.$$

At each stage n of the regression (13) values, $a_{0}$, $a_{1}$, and $a_{2}$ were calculated using the stated norms $∥R_{1}{∥}_{2},$ $∥R_{2}{∥}_{2},\ldots ,∥R_{n}{∥}_{2},$ of the existing reconstruction images in the following way: on the outer level, we used a procedure of ternary search through the values of the parameter $a_{1}$, then using a simple linear least-squares fitting, with a fixed value of $a_{1}$, the values of $a_{0}$ and $a_{2}$ were obtained. The values determined on each stage were used to extrapolate the value of $∥R_{T}{∥}_{2}$. After all, we needed to estimate the Radon invariant $S\left(R_{T}\right)$ to implement the stopping rule $N_{\Delta }^{S-\mathrm{RSRE}}\;$(8). The values of the Radon invariant did not differ notably from the values $S\left(R_{1}\right),S\left(R_{2}\right),\ldots ,S\left(R_{n}\right)$ and from the sums of elements in each projection $S\left(x_{1}\right),S\left(x_{2}\right),\ldots ,S\left(x_{n}\right)$. All currently available projections were used to calculate the noise reduction,

(14) $$S\left(R_{T}\right)\approx \frac{1}{n}\overset{n}{\underset{i=1}{\sum }}S\left(x_{i}\right).$$

Below, we present the evaluation of the monitored tomographic reconstruction process with the presented framework.

## 4. Results and Discussion

**Figure 4:**
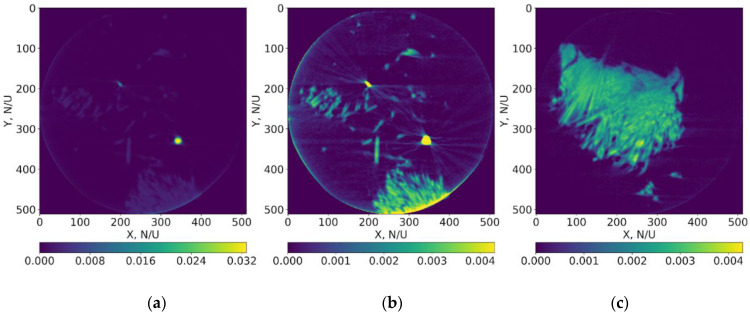
Examples of reconstructed sections with a maximal difference in their morphology: (**a**) is 175th slice presented in the full interval of reconstructed values, (**b**) is 175th slice with limited interval to pronounce the object and (**c**) is 260th slice.

In our simulation experiment, we used five different 2D slices of the object, choosing slices with different morphologies. The numbers of the slices are 124, 136, 175, 243, and 260. Two slices (numbers 175 and 260, from five slices used in the experiment) reconstructed from the full set of the collected projections are presented in Figure 4. An FBP algorithm was used for partial reconstruction. As slice 175 contains the gold particles used as fiduciary markers, we present this slice in two different intensity scales (Figure 4a,b). For 4a and 4b images (260 and 175 slices, respectively), the dynamic intervals of the pixel intensity values are the same, i.e., they correspond to the sample intensities interval.

**Figure 5:**
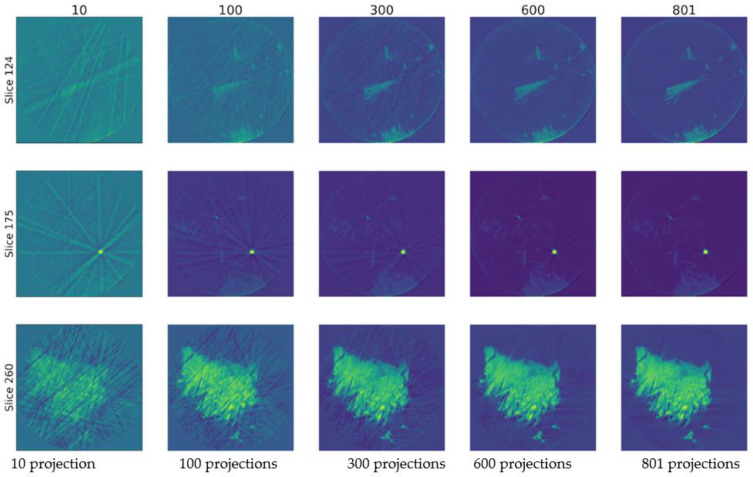
Reconstructed slices with 10, 100, 300, and 600 randomly acquired projections. Right column is the reconstructed slices with full set of collected projections. It is given for reference.

Examples of partial reconstructions (three chosen slices from five slices used in the experiment: 124, 175, and 260) are presented from top to bottom in Figure 5. The number of projections used for the reconstruction are 10, 100, 300, 600, and 801, respectively. 

**Figure 6:**
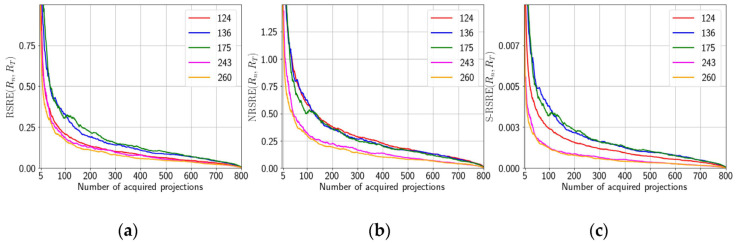
Convergence graphs for different error functions for five chosen slices: (**a**) is for RSRE, (**b**) is for NRSRE and (**c**) is for *S*-RSRE.

We present the error functions RSRE (Figure 6a), NRSRE (Figure 6b), and S-RSRE (Figure 6c) to confirm our conclusion about different convergences to the last result.

**Figure 7:**
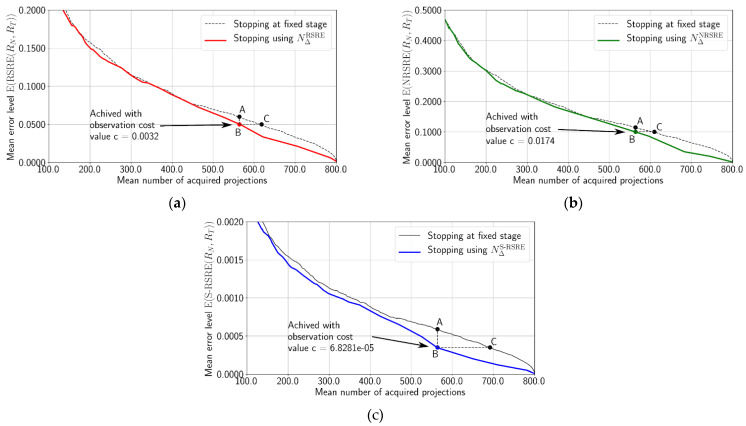
Expected performance and baseline profiles for three estimation errors: (**a**) is for RSRE, (**b**) is for NRSRE and (**c**) is for *S*-RSRE.

We evaluated the constructed stopping rules. In Figure 7, the expected performance profiles [21] of the rules for RSRE, NRSRE, and *S*-RSRE are presented. To obtain the points of the gray baseline plots, we fixed the projection number and calculated the mean error function value for all reconstructed slices. The stopping rule performance profile is obtained by fixing the value of the cost *c*. For each slice, the monitored reconstruction was done. To obtain the abscissa of the point (*X*-axis value), we calculated the mean number of the projections, and to obtain the *Y*-axis value, we calculated the mean error value achieved at stopping time. In all cases, the use of stopping rules (blue, green, and red lines) demonstrates the gain in the number of used projections.

Below, we consider in detail Figure 7a, describing the points A, B, and C. Point A of the baseline corresponds to the following moment. Exactly 550 projections were used for the reconstruction of each slice. Then, the mean RSRE error was calculated. The mean error level is 0.06. The cost value c = 0.0032 for the same number of the projections on average for all slices in the monitored reconstruction case (red line) corresponds to point B. The mean error level is 0.05. This level on the reference plot (baseline) corresponds to point C. The triples of points on the residual two graphs are interpreted similarly.

The profiles are constructed by placing the mean error level of partial reconstructions against the mean number of projections required before the stopping condition is met, while varying the cost parameter c. The mean error level is expressed in terms of the error function value $\epsilon \left(R_{n},\theta \right)$ averaged through the slices. As a reference, we plot the mean error level achieved by reconstruction of the slice with a fixed number of the projections.

## 5. Conclusions

A monitored tomographic reconstruction process that performs the reconstruction procedure not only after the completion of the measurement protocol but simultaneously to the acquisition of the individual radiographies was proposed. The data acquisition for each projection or group of the projections is interspersed by fast [22] image reconstruction. We demonstrated a new approach for nano-XCT data for a novel transition-metal-based materials system: MoNi_4_ electrocatalysts anchored on MoO_2_ cuboids aligned on Ni foam (MoNi_4_/MoO_2_@Ni). Applying the stopping rules to the monitored tomographic reconstruction process decreases the average level of error with a fixed average number of projections. This effect is demonstrated for the three stopping rules based on the plotting of the expected performance profiles.

The proposed new protocol for image data acquisition (“design of experiment”) and advanced data analysis reduces time-to-data for nano-XCT studies of nanomaterials by more than an order of magnitude, while maintaining sub-100 nm resolution and high image quality. In addition, the reduced dose is an advantage for the study of radiation–sensitive biological and organic materials. Finally, the advanced protocol is also applicable to synchrotron radiation 3D imaging studies, with particular benefits for in situ and operando studies of kinetic processes in materials.

## Data Availability

Original measurement data are available at Fraunhofer IKTS Dresden, Germany.
